# Diagnostic Value of Positron Emission Tomography Combined with Computed Tomography for Evaluating Critically Ill Neurological Patients

**DOI:** 10.3389/fneur.2017.00033

**Published:** 2017-02-14

**Authors:** Knut Kurt William Kampe, Roman Rotermund, Milena Tienken, Götz Thomalla, Marc Regier, Susanne Klutmann, Stefan Kluge

**Affiliations:** ^1^Department of Intensive Care, University Medical Center Hamburg-Eppendorf, Hamburg, Germany; ^2^Department of Neurosurgery, University Medical Center Hamburg-Eppendorf, Hamburg, Germany; ^3^Nuclear Medicine, Department of Diagnostic and Interventional Radiology and Nuclear Medicine, University Medical Center Hamburg-Eppendorf, Hamburg, Germany; ^4^Department of Neurology, University Medical Center Hamburg-Eppendorf, Hamburg, Germany; ^5^Diagnostic and Interventional Radiology, Department of Diagnostic and Interventional Radiology and Nuclear Medicine, University Medical Center Hamburg-Eppendorf, Hamburg, Germany

**Keywords:** FDG-PET/CT, paraneoplastic, cerebral vasculitis, sepsis, critical care

## Abstract

**Purpose:**

^18^F-fluorodeoxyglucose positron emission tomography combined with computed tomography (FDG-PET/CT) is a promising new tool for the identification of inflammatory, infectious, and neoplastic foci. The aim of our work was to evaluate the diagnostic value of FDG-PET/CT in patients treated on a neurological/neurosurgical ICU or stroke unit.

**Methods:**

We performed a single-center, 10-year, retrospective evaluation of the value of FDG-PET/CT in critically ill adult patients with severe neurological disease.

**Results:**

42 patients underwent FDG-PET/CT. Of these, 15 were ventilated and 10 were under vasopressor support. We identified four indications for performing FDG-PET/CT: (1) excluding a paraneoplastic etiology in an otherwise unexplained encephalitis, encephalopathy or neuropathy, (2) detecting a large-vessel vasculitis in patients with ischemic stroke, (3) detecting an infectious focus in sepsis, and less frequently (4) evaluating cerebral metabolism. In 22 patients who were evaluated for an unknown malignancy, 5 scans revealed either a previously unknown tumor or unknown metastases of a previously treated malignancy. Of 11 patients investigated for large-vessel vasculitis, 2 showed an inflammation of arteries supplying the brain. Of six sepsis cases, FDG-PET/CT identified an infectious focus in four.

**Summary:**

We found FDG-PET/CT to be a helpful tool in critically ill neurological patients. The results of the FDG-PET/CT had direct therapeutic consequences in the 12 true-positive cases. In 24 of the 29 negative cases, FDG-PET/CT helped exclude alternative diagnoses and/or influenced therapy. Our findings demonstrate the feasibility and diagnostic benefit of FDG-PET/CT in this group of patients.

## Introduction

In recent years, ^18^F-fluorodeoxyglucose positron emission tomography (FDG-PET) has become a recognized and well-researched imaging modality for the evaluation of neoplastic, inflammatory, and infectious disorders (1). Increased FDG uptake indicates increased intracellular glucose metabolism. FDG may accumulate in malignant cells as well as in cells involved in inflammatory and infectious processes, such as macrophages. Recently, PET technology has been combined with computed tomography (CT) scanning in a single device. Hybrid imaging by FDG-PET/CT offers considerable advantages over regular CT by providing anatomical information in addition to detecting areas with increased metabolism. Several studies have documented the important role of FDG-PET/CT in detecting unknown malignancies (2), detecting large-vessel vasculitis, (3) and diagnosing patients with fever of unknown origin (4).

To our knowledge, there are no previously published reports specifically on the use of FDG-PET/CT to identify underlying pathologies in critically ill neurological patients. Furthermore, data on the use of FDG-PET/CT in critically ill patients are scarce, and it has only been used in cases of sepsis or septic shock (5, 6). The aim of our 10-year, retrospective analysis was to discover which questions were addressed by FDG-PET/CT in our set of critically ill, neurological patients and to determine whether FDG-PET/CT had been helpful in identifying the relevant underlying pathology.

## Materials and Methods

### Study Design

This retrospective, observational study included all critically ill, adult patients treated on the neurological ICU, neurosurgical ICU or stroke unit at the University Medical Center Hamburg-Eppendorf from March 2006 to May 2015 who had undergone FDG-PET/CT for any reason. The study complied with the requirements of the local Clinical Institutional Review Board and was performed in accordance with the Declaration of Helsinki.

We extracted the following information from the hospital’s record system (Integrated Care Manager, Dräger Medical, Germany and Soarian Clinicals, Cerner, USA, Inc.): demographics, diagnosis on admission, diagnosis on discharge (from ICU/hospital), duration of stay in ICU/hospital, diagnostic procedures and treatment prior to FDG-PET/CT, transport-related complications, clinical course and changes in clinical management after having results from FDG-PET/CT, and survival. FDG-PET/CT was requested by the attending intensivists at their discretion when they considered clinical signs and/or laboratory and/or imaging findings to be inconclusive.

### ^18^F-Fluorodeoxyglucose Positron Emission Tomography Combined with Computed Tomography

All patients were fasted for at least 4 h before scanning; parenteral nutrition and infusions containing glucose were also discontinued during that period. Blood glucose concentration was checked every hour prior to the administration of 350 MBq of 18 F-FDG, which was only performed at glucose levels below 200 mg/dl. All patients were fully monitored during transport and the procedure and were accompanied by a qualified intensive care physician and a registered nurse. FDG-PET/CT was performed with a hybrid PET/CT scanner (Gemini GXL10, Philips, Best, Netherlands). The CT part of the examination included the use of intravenous contrast medium. All patients without elevated levels of serum creatinine received 100–200 ml intravenous contrast agent (Imeron 300, Bracco Imaging Konstanz, Germany) 90 s before starting the CT scan. The scan took between 30 and 45 min, including positioning the patient. Whole-body images were acquired from head to leg. All PET scans were performed in 3-dimensional acquisition mode. Emission images were acquired for 1.5 min per position at head, thorax, and abdomen and 60 s at the legs (effective axial field of view: 90 cm). The acquisition parameters were as follows: 120 kV, 150 mA, slice thickness 5 mm, no gap, pitch 0.9, rotation time 0.74 sec, matrix 512 × 512. CT data extrapolated to 511 keV were used for low noise attenuation correction of PET data and for subsequent co-registration with attenuation-corrected PET images. PET, CT, and fused FDG-PET/CT images were provided for review and displayed in axial, coronal, and sagittal planes. The PET data were available as uncorrected and attenuation-corrected images and also as a rotating maximum-intensity projection.

### Interpretation and Analysis of Contrast-Enhanced FDG-PET/CT Images

An interdisciplinary team, including one board certified radiologist and one board certified nuclear medicine physician, who had knowledge of the patient’s clinical history, evaluated all scans. Initially, PET and CT images were read independently: PET images were evaluated by two specialists in nuclear medicine (one of them a senior consultant in nuclear medicine) and CT images were evaluated by two physicians from the Department of Radiology (one of them a senior consultant). Afterward, the results of both parts of the investigation were reviewed in a consensus conference of all four physicians resulting in a final written report for each FDG-PET/CT investigation.

A focus of increased FDG uptake was considered abnormal if it was elevated above the regional background in localizations outside the expected physiological FDG-distribution. The definite clinical diagnosis, which served as the reference standard, was made retrospectively taking all available clinical information into consideration. These sources of information included clinical course, physical examination, laboratory, microbiology and histology results as well as intraoperative findings, electrophysiological examinations, and imaging results other than the FDG-PET/CT. The FDG-PET/CT results were then compared with the final clinical diagnosis and interpreted as true positive (TP) or false positive (FP) and true negative (TN) or false negative (FN).

The FDG-PET/CT scan was considered:

- TP—if FDG-PET/CT results demonstrating a localized disease process were confirmed by further investigations as the cause of the disorder under investigation;
- FP—if FDG-PET/CT results demonstrating a localized disease process could not be confirmed as being the cause of the disorder under investigation;
- TN—if FDG-PET/CT produced a normal scan and both clinical follow-up, and further investigations excluded the suspected diagnosis for the disorder under investigation;
- FN—if FDG-PET/CT produced a normal scan, but the suspected diagnosis was confirmed by other diagnostic procedures.

We also determined whether the FDG-PET/CT results were helpful to the clinician, e.g., were essential for making a diagnosis or excluding a diagnosis and thus had therapeutic consequences. Depending on the question addressed, TP scans *and* TN scans could be considered to be “helpful.”

Reference values concerning the established sensitivity and specificity of FDG-PET/CT have been published for detecting an underlying malignancy in patients with clinically suspected neurological paraneoplastic syndromes (PNSs) (7), detecting cerebral metastases (8, 9), detecting a large-vessel vasculitis (10), and detecting the etiology of fever of unknown origin (11), as well as for determining characteristic spatial patterns of brain metabolism in various subtypes of dementia (12).

### Statistical Analysis

Continuous variables are provided as means (±SD) for normally distributed data, or as medians (with the range) for non-normally distributed data. They were calculated using Excel (Microsoft, USA).

## Results

### Patient Characteristics

During the study period, 42 FDG-PET/CT scans were performed on critically ill adult patients: 26 were referred from the neurological ICU, three from the neurosurgical ICU, and 13 from the stroke unit. Demographics, mortality rates, and admission diagnoses of the study population are shown in Table 1.

**Table 1:** **Demographics, mortality rates, admission diagnosis**.

No. of patients	42
Mean age of ALL patients	56.1 ± 15.5 years
With suspected malignancy	55.4 ± 15.0 years
With suspected vasculitis	48.8 ± 11.0 years
With suspected infectious focus	71.3 ± 10.4 years
Mean time in ICU	23.6 ± 16.9 days
Median interval between ICU admission and PET/CT	13 days (range 1–43)
Patients ventilated at time of scan	15 (35.7%)
Patients on vasopressor support at time of scan	10 (23.8%)
Mortality rate	1 (2.4%)
Admission diagnosis	
Ischemic stroke	10 (23.8%)
Subarachnoid hemorrhage	3 (7.1%)
Encephalitis/meningitis	8 (19.0%)
Encephalopathy	5 (11.9%)
Seizures	5 (11.9%)
Neuropathy	6 (14.3%)
Infection of unknown origin	5 (11.9%)

No complications occurred during transportation to or from the ICU and the Department of Nuclear Medicine. Adverse reactions to the tracer or the intravenous contrast agents were not observed, and the investigation procedure was well tolerated by all patients. Hospital survival rate was 97.6% (41 of 42 patients).

We found four indications for performing an FDG-PET/CT in these patients: (1) excluding a malignancy as the cause of neurological symptoms, (2) detecting a large-vessel vasculitis of arteries serving the brain, (3) detecting a septic focus, and less frequently (4) evaluating cerebral metabolism.

### Diagnostic Value of FDG-PET/CT Results

All diagnostic tests performed before FDG-PET/CT, the final diagnosis, and correlation with FDG-PET/CT findings (42 scans) are shown in Tables A–C in Supplementary Material. Pathological FDG accumulations were found in 13 of 42 (31%) of PET/CT scans, of which 12 (29%) were TPs. These TPs were observed most frequently in the group of patients with suspected unknown infectious focus followed by the group with suspected malignancy and finally in those with suspected vasculitis (67, 23, and 18%, respectively). Negative results were obtained in 29 cases (69% of FDG-PET/CT scans). None of these findings were considered FNs. This represents an overall accuracy of 97.6%. Table 2 shows the distribution of TPs (12), FPs (1), TNs (29), and FNs (0) in relation to the suspected diagnosis (i.e., suspected malignancy, suspected vasculitis, suspected infectious focus, or altered cerebral metabolism).

**Table 2:** **Diagnostic value of an FDG-PET/CT scan**.

Indication	True positive	False positive	True negative	False negative	PET-CT considered helpful	PET-CT not helpful
Detecting a malignancy (22 patients)	5 (23%)	1 (4%)	16 (73%)	0	21 (96%)	1 (4%)
Detecting a vasculitis (11 patients)	2 (18%)	0	9 (82%)	0	8 (73%)	3 (27%)
Detecting a septic focus (6 patients)	4 (67%)	0	2 (33%)	0	4 (67%)	2 (33%)
Evaluating cerebral metabolism (3 patients)	1 (33%)	0	2 (67%)	0	3 (100%)	0

We also recorded whether the clinician deemed the FDG-PET/CT “*helpful*.” This was usually the case when the scan was essential for making a diagnosis or had therapeutic consequences. In this context, all TP results were considered helpful. However, depending on the question addressed, TN scans could also be “*helpful*,” and this was the case in 24 of the 29 negative investigations in which the PET/CT result helped exclude alternative diagnoses and/or influenced therapy. Negative results were especially helpful when excluding a malignancy or a large-vessel vasculitis as the cause of neurological symptoms and rather unhelpful when searching for infectious foci in persisting sepsis. Overall, PET/CT was considered helpful in 86% of cases.

### Patients with Suspected Malignancy

**Figure 1:**
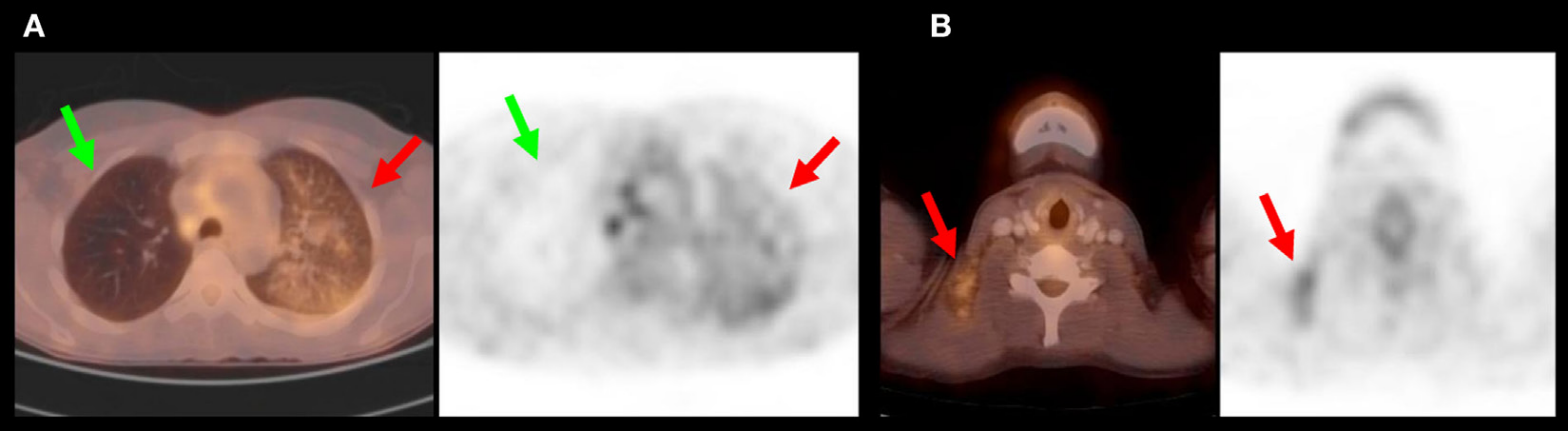
**This 36-year-old woman had a thrombosis of her left leg due to a coagulopathy and suffered multiple embolic ischemic strokes**. Neither ultrasound of the carotid arteries, cardiac ultrasound, nor cerebral angiography revealed any causative pathology. **(A)** PET/CT revealed a diffuse, blotchy enhancement of the left lung suggestive of a bronchoalveolar carcinoma (red arrows). Green arrows depict the healthy right lung. The PET/CT overlay is shown on the left side, with the conventional PET-scan on the right. **(B)** Multiple suspicious lymph nodes were identified in the neck. A biopsy revealed an adenocarcinoma.

The single most important reason for performing an FDG-PET/CT scan was detecting a suspected malignancy. This was the reason for 22 (52%) of the scans. In 12 of these patients, the clinician suspected a paraneoplastic encephalitis or encephalopathy, in 6 a paraneoplastic neuropathy, in 3 directly a tumor or metastasis, and in 1 patient a paraneoplastic coagulopathy (see Figure 1). Five scans (22.7%) revealed either a previously unknown tumor or metastasis (TP). Detection of a metastasis could indicate the recurrence of previously treated malignancy, a novel tumor or the presence of a cancer of unknown origin. In four patients, FDG-PET/CT was crucial for the detection of the malignancy. In the other patient, the PET/CT confirmed a suspected malignancy.

In one case, a suspected malignancy was not confirmed after extensive workup (FP). In the 16 negative cases, the FDG-PET/CT was considered helpful in excluding the differential diagnosis of a tumor.

### Patients with Suspected Large-Vessel Vasculitis

**Figure 2:**
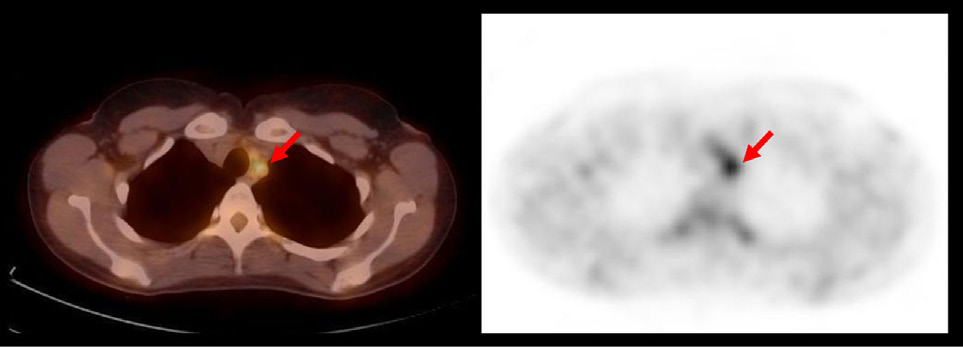
**This 38-year-old woman presented with severe headaches**. MRI revealed a swollen right hemisphere and impaired cerebral perfusion. Treatment with steroids was initiated. PET/CT revealed an enhancement of the supra-aortic arteries suggestive of a large-vessel vasculitis (red arrows). Takayasu arteritis was diagnosed.

The second most important reason for performing an FDG-PET/CT scan was to demonstrate or exclude a large-vessel vasculitis (11 patients, 26% of all cases). Two of the 11 patients (18.2%) displayed inflammation of large vessels supplying the brain, indicative of a Takayasu vasculitis (TP) (see Figure 2).

In three cases, a negative PET/CT was considered helpful as the scan demonstrated no persisting large-vessel vasculitis under immunosuppressive therapy (TN after therapy, helpful). In a further three cases, the diagnosis of a vasculitis was rejected after PET/CT in conjunction with other studies did not demonstrate any pathology (TN, helpful).

In the three remaining cases, PET/CT helped exclude a large-vessel vasculitis, but based on further workup, a diagnosis of a medium/small vessel vasculitis was made. In two of these cases, a conventional angiography was decisive. In the third case, the clinical course was highly suggestive of a vasculitis. In all three patients immunosuppressive therapy was initiated and maintained. In these three cases, PET/CT was not helpful in determining the underlying pathology (TN, not helpful).

### Patients with Suspected Infectious Focus

**Figure 3:**
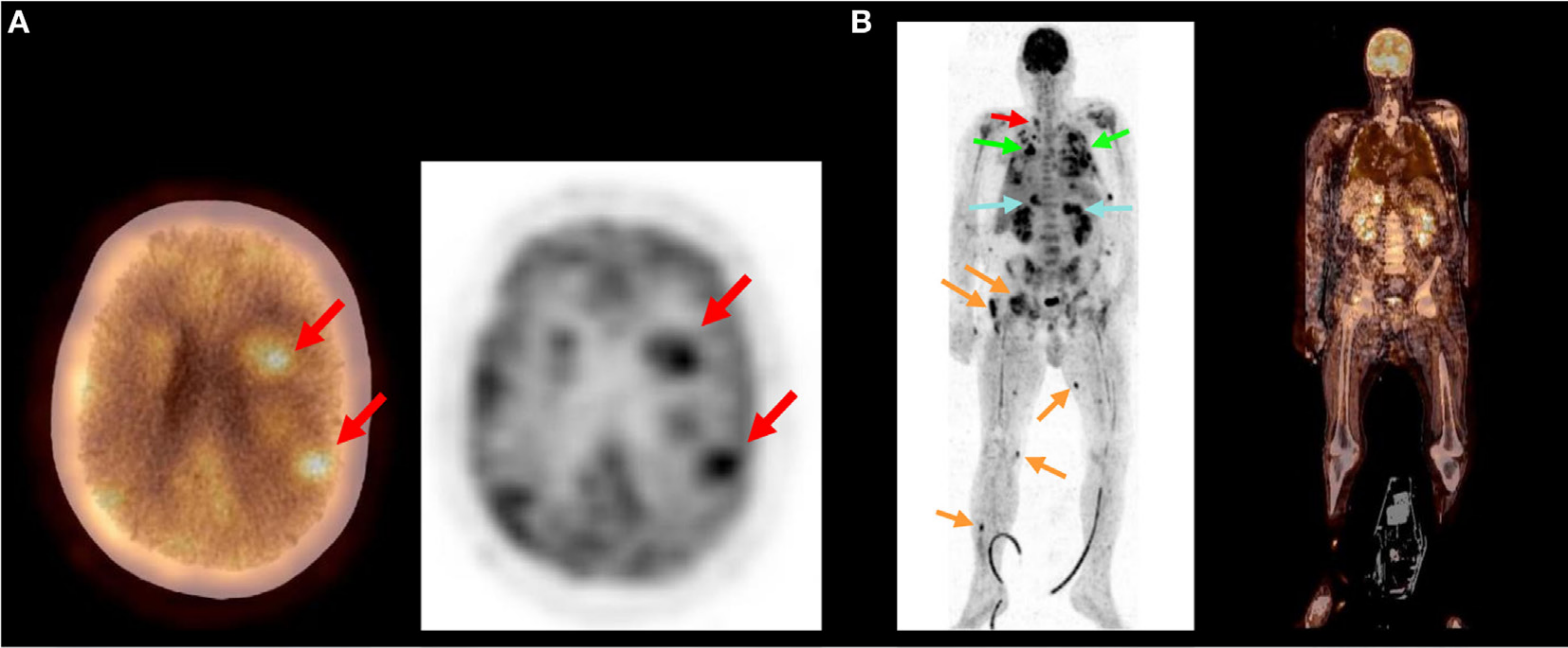
**This 57-year-old man presented with fever and shortness of breath**. Antibiotic treatment was initiated. Shortly, he developed right sided hemiparesis and confusion. Cultures remained sterile. **(A)** PET/CT revealed multiple cerebral and cerebellar abscesses. **(B)** Further abscesses were identified throughout the body: at the side of the thyroid gland (red arrow), within the lung (green arrows), liver, and both adrenal glands (blue arrows) as well as multiple foci within muscles (orange arrows). A nocardiosis was suspected. *Nocardia farcinia* was eventually cultured from a bronchoalveolar lavage confirming the diagnosis.

Detecting the inflammatory focus in patients with sepsis, in whom the etiology could not be determined otherwise, was a less common indication for performing an FDG-PET/CT scan in our case series of neurological patients (six subjects, 14% of all cases). Nevertheless, when performed, relevant pathological results were demonstrated in 66.7% of cases (four of six patients). An example in which determining the extent of the infection was helpful is shown in Figure 3.

In cases of sepsis, only TP scans were considered “helpful” by the clinician. One patient (TP in PET/CT) died as a result of septic shock with multiple organ failure due to a spondylodiscitis.

### Patients with Altered Cerebral Metabolism

Altered cerebral metabolism refers to changes in FDG uptake by brain tissue itself in contrast to FDG uptake by cells that may have invaded the brain such as tumor cells or inflammatory cells. Evaluating cerebral metabolism was a relatively rare reason for performing an FDG-PET/CT scan in the study population (3 subjects, 7% of cases). The first patient displayed rapidly progressive cognitive decline, but the PET/CT was normal. No evidence for a neoplastic or inflammatory lesion was found. A neurodegenerative disease was excluded. The final diagnosis was a normal pressure hydrocephalus.

The second patient suffered a cerebellar hemorrhage. Thorough clinical and neurophysiological investigations could not determine whether the patient was awake and conscious. The PET/CT demonstrated a completely normal supratentorial activity. A diagnosis of locked-in syndrome was made.

The third patient suffered from intractable focal seizures. The FDG-PET/CT demonstrated focally increased brain activity. Seizures ceased after neurosurgical extraction of the epileptic focus. In all three cases, the PET/CT was considered helpful by the clinician.

### Additional Findings

Twenty-seven of the 42 scans (64%) revealed additional findings of which 3 (11%) had therapeutic consequences. These were a thrombosis adjacent to a central venous catheter (the catheter was removed), a thrombus in the right jugular vein (anticoagulation was increased), a subdural hygroma not present on a previous CT scan (the hygroma was surgically drained).

## Discussion

Our findings show that FDG-PET/CT is useful for the diagnostic work-up of critically ill neurological patients. The FDG-PET/CT scan was considered to be “helpful for diagnosis or treatment” in 36 of 42 patients (86%) by the treating clinician. Positive (33%) as well as negative (67%) findings contributed to this opinion. The ratio of positive to negative findings was clearly related to the diagnosis under investigation.

The largest group in our sample was patients with suspected PNS. Previous research has suggested that the yield of FDG-PET/CT may be rather low when investigating PNS (13). Bannas et al., using combined PET/CT, identified malignancy as the underlying cause of suspected PNS in only 8.7% of cases. In our sample of critically ill neurological patients, the yield was higher (22.7%). This number is closer to the 19% reported by other investigators (14). In our study population, a large proportion had already been investigated extensively before the decision was made to perform a PET/CT scan. The cost of getting these critically ill patients into the scanner may have led to a more rigorous selection, explaining the higher yield compared with other studies.

In our series, 22 patients were investigated for an unknown malignancy. FDG-PET/CT revealed a previously unknown tumor in two cases and a recurrence of a previously treated tumor in three further cases. PET/CT has been shown to be better at detecting unknown malignancies than other modalities such as contrast-enhanced CT (CECT) (15). Nevertheless, in our study, FDG PET-CT was mostly performed as an add-on after inconclusive or negative results from regular investigations attempting to detect a malignancy (e.g., CECT, abdominal ultrasound, and search for paraneoplastic antibodies). In two cases, PET/CT revealed a tumor despite extensive prior workup including CECT. In a further case, CECT had indicated a spinal tumor, and PET/CT was performed to provide more detail and search for further metastases. In only nine cases, an FDG/CT was performed without prior CECT workup. This decision was made by the responsible physician. Reasons for this approach were a high suspicion of malignancy, avoiding repeat investigations in critically ill patients, and reducing the radiation exposure from CECT plus PET/CT. In two of these nine cases, in which FDG-PET was performed without prior CECT, a malignancy was detected (22%).

The cause of PNS was identified as a bronchial carcinoma (two cases), a breast carcinoma (two cases), and a prostate carcinoma (one case). PNSs are associated most frequently with lung cancer (16), but PNS may also occur in other cancers such as breast cancer (17) and prostate cancer (18).

We found FDG-PET/CT helpful for detecting or excluding a large-vessel vasculitis as well as for monitoring disease activity in patients with established large-vessel vasculitis. In the two cases with positive findings, the final diagnosis was a Takayasu arteritis (TA). The clinical value of FDG-PET in detecting TA is well established (19). In addition, PET/CT may be helpful in monitoring the activity of a large-vessel vasculitis after treatment. As previously described, PET/CT may reliably detect early changes in the disease and identify non-responders to therapy (10, 20). In three such cases with previously established large-vessel vasculitis, the negative PET/CT finding was considered helpful in evaluating treatment success by the clinician.

It is important to point out that an inflammation of middle-sized or small vessels may escape detection in an FDG PET/CT scan. Determining the presence of a vasculitis restricted to the small arteries of the central nervous system is extremely difficult (21). Often a combination of investigations including conventional angiography, MRI, cerebrospinal fluid examination, and possibly even histologic confirmation after a biopsy is required for the diagnosis of a primary angiitis of the central nervous system. In our set of patients, a conventional angiography was decisive in two cases in which PET/CT had yielded negative results. In addition to technical investigations, a comprehensive and systematic review of the clinical course is paramount for the final diagnosis of a vasculitis (22). In one of our cases, the clinical course led to a decision that a cerebral vasculitis must be present despite inconclusive or negative findings in all investigations including PET/CT.

Detecting the source of an infection in patients with sepsis was a less frequent reason for performing FDG-PET/CT in our set of patients. Nevertheless in the six cases, in which investigations were requested with this question in mind, they had considerable urgency due to the fact that prompt and adequate therapy is crucial for survival in sepsis (23). PET/CT was successful in identifying the source of an infection in a large proportion of cases, comparable to the results of Kluge et al. (5) (67% here compared to 61% in the previous study). The results often indicated specific therapeutic measures or changes in therapy. Interestingly, the only recorded fatality occurred in this group. When searching for the infectious focus in a patient with persistent sepsis, only TP scans were considered helpful by the clinician.

Evaluating cerebral metabolism was a relatively rare indication for performing an FDG-PET/CT in our population of critically ill neurological patients. One patient was investigated to clarify the nature of a rapidly progressing dementia. Brain FDG-PET can show characteristic spatial patterns of brain metabolism which are helpful in differentiating subtypes of dementia and thus may be helpful in making a diagnosis and in initiating appropriate management (12). However, FDG-PET/CT did not yield a pathological finding specific to a particular type of dementia in this case. A second patient underwent PET/CT scans to identify an epileptic focus. FDG-PET has been used to localize epileptic foci for some time (24). After characterization of the epileptic focus, this patient proceeded to surgical treatment. One patient received a PET/CT scan to determine whether he had locked-in syndrome or was in a persistent vegetative state after a cerebellar hemorrhage. Extensive clinical monitoring had yielded inconclusive results. Recent studies have suggested using technology-based assessments including PET in this challenging group of patients with disorders of consciousness (25–27). FDG-PET/CT demonstrated a regular supracortical pattern in this patient leading to the conclusion that he had locked-in syndrome. Although investigating cerebral metabolism with FDG-PET/CT is possible in critically ill neurological patients, it is a rather rare indication which can usually be postponed until the patient is more stable.

In summary, FDG-PET/CT scanning appears to be a valuable diagnostic tool in this selected population of critically ill neurological patients when extensive prior diagnostic work-up has been negative or inconclusive. Nevertheless, the difficulties in performing this diagnostic procedure in such a cohort of patients should be mentioned. One of these is that transporting critically ill patients from the ICU or stroke unit to the department of nuclear medicine puts these patients at risk, especially when they require ventilator or vasopressor support (28, 29). However, no adverse events related to transportation or the imaging itself were observed in our case series. Furthermore, the investigation requires substantial resources in terms of both time and personnel in addition to being rather expensive (30). These considerations need to be weighed against the diagnostic benefit in this population with high morbidity.

The interpretation of the results of this study is limited by the retrospective design and the relatively small number of patients per subgroup. However, to our knowledge, this is the first study addressing the value of FDG-PET/CT scans specifically in critically ill neurological patients.

## Ethics Statement

The study complied with the requirements of the local Clinical Institutional Review Board (Ethik-Kommission der Ärztekammer Hamburg). For this type of retrospective study, formal consent is not required. All procedures performed in this study involving human participants were in accordance with the ethical standards of the institution and with the 1964 Helsinki declaration and its later amendments.

## Author Contributions

KK: concept for the paper, collected and evaluated the data, and wrote the paper. RR: collected and evaluated the data. MT: contributed to evaluation and presentation of the PET data in the paper. GT: clinical input to the patients involved, correlation between clinical presentation and PET/CT scans, and contributed in a major way to the revision of the paper. MR: radiological evaluation of the CT scans, and correlation with PET-scans. SusanneK: evaluation of the PET-Data, correlation with CT scans and clinical course, and conceptualization of the project. StefanK: idea of the project, conceptualization of the project, and major contribution to the revision of the paper.

## Conflict of Interest Statement

The authors declare that the research was conducted in the absence of any commercial or financial relationships that could be construed as a potential conflict of interest.
