# Supplementary material for: Diagnostic Value of Positron Emission Tomography Combined with Computed Tomography for Evaluating Critically Ill Neurological Patients
Source: Front Neurol. 2017 Feb 14;8:33. doi: 10.3389/fneur.2017.00033 (PMC5306377; doi:10.3389/fneur.2017.00033)
Supplement: Supplementary file 1 [file Table_1.DOC]

**Diagnostic value of Positron Emission Tomography combined with Computed Tomography for evaluating critically ill neurological patients**

Knut Kampe^1^, Roman Rotermund^1,2^, Milena Tienken^3^, Götz Thomalla^4^, Marc Regier^5^, Susanne Klutmann^3^, Stefan Kluge^1^,

**Supplemental Material (to be presented online):**

**Table A Patients with suspected paraneoplastic disease**

Diagnostic tests before FDG-PET/CT, final diagnosis and correlation with FG-PET/CT:

| **Pat. No.** | **Age/ Sex** | **Days  on ICU/ stroke** | **MRI: Brain** | **CT: Brain/ Chest/ Abd.** | **US abd.** | **EEG** | **CSF** | **NCS** | **Paraneoplastic/ or auto-antibodies** | **Question addressed by FDG-PET/CT:**  **→Result** | **Confirmed Diagnosis** | **PET CT** |
| --- | --- | --- | --- | --- | --- | --- | --- | --- | --- | --- | --- | --- |
| 1 | 57/F | 20 | **+** | **+/+/+** | **+** | **+** | **+** | **-** | negative | Encephalitis: Evidence for recurrence of mammary carcinoma?  → Demonstration of multiple metastases | Paraneoplastic encephalitis Mammary carcinoma | TP |
| 2 | 60/M | 7 | **+** | **-/+/+** | **+** | **+** | **+** | **-** | Anti-NMDA receptor | Encephalitis: Paraneoplastic etiology?  → No evidence for malignancy | Anti-NMDA receptor encephalitis | TN |
| 3 | 44/F | 36 | **+** | **+/-/-** | **-** | **+** | **+** | **-** | Anti-K^+^ channels | Encephalitis: Paraneoplastic etiology?  → No evidence for malignancy | VGKC-AB Limbic encephalitis | TN |
| 4 | 65/F | 5 | **+** | **-/+/+** | **-** | **+** | **+** | **-** | Anti-Kv 1.1 and 1.6 | Encephalitis: Paraneoplastic etiology?  → No evidence for malignancy | Suspected Prion disease; Sporadic Fatal Insomnia | TN |
| 5 | 39/M | 49 | **+** | **-/+/+** | **-** | **+** | **+** | **-** | Anti-NMDA receptor | Encephalitis: Paraneoplastic etiology?  → No evidence for malignancy | Anti-NMDA receptor AB limbic encephalitis | TN |
| 6 | 19/F | 38 | **+/** +MRI abd. | **-/-/**- | **+** | **+** | **+** | **-** | Anti-NMDA receptor | Encephalitis: Paraneoplastic etiology?  → No evidence for malignancy | Anti-NMDA receptor encephalitis | TN |
| 7 | 68/M | 29 | **+** | **+/+/-** | **-** | **-** | **+** | **+** | negative | Brainstem encephalitis: Paraneoplastic etiology?  → No evidence for malignancy | Bickerstaff encephalitis | TN |
| 8 | 75/F | 33 | **+** | **+/+/+** | **-** | **+** | **+** |  | negative | Encephalitis: Paraneoplastic etiology?  → No evidence for malignancy | Tuberculous Meningitis | TN |
| 9 | 68/F | 12 | **+** | **+/-/-** | **+** | **+** | **+** | **-** | negative | Encephalopathy and neuropathy: Paraneoplastic etiology?  → suspicious spinal lesion | Mammary carcinoma with spinal metastases | TP |
| 10 | 72/M | 73 | **+** | **+/-/-** | **-** | **+** | **+** |  | negative | Encephalopathy: Paraneoplastic etiology?  → Demonstration of Metastasis of known prostate cancer | Paraneoplastic encephalopathy metastatic prostate carcinoma | TP |
| 11 | 66/M | 59 | **+** +MRI Spine | **+/+/+** | **+** | **+** | **+** | **+** | negative | Encephalopathy: Paraneoplastic etiology?  → No evidence for malignancy | Suspected autoimmune meningoencephalitis | TN |
| 12 | 62/F | 40 | **+** | **+/-/-** | **+** | **+** | **+** | **-** | negative | Encephalopathy: Paraneoplastic etiology?  → No evidence for malignancy | Impaired consciousness of unknown etiology | TN |
| 13 | 47/F | 34 | **+/** +MRI Spine | **+/+/+** | **+** | **-** | **+** | **+** | Anti-HU | Neuropathy: Paraneoplastic etiology?  → Demonstration of lymph node metastases | Paraneoplastic axonal neuropathy small cell bronchial carcinoma | TP |
| 14 | 70/F | 16 | **-** | **-/+/-** | **+** | **-** | **+** | **+** | negative | Neuropathy, mammary carcinoma: Paraneoplastic etiology?  → No evidence for malignancy | Acute axonal neuropathy Paraneoplastic disease excluded | TN |
| 15 | 50/F | 14 | **+/** +MRI Spine | **-/-/-** | **-** | **-** | **+** | **+** | negative | Neuropathy: Paraneoplastic etiology?  → No evidence for malignancy | Autoimmune sensomotoric neuropathy Sjögren Syndrome | TN |
| 16 | 52/M | 27 | **+** +MRI Spine | **-/-/-** | **-** | **+** | **+** | **+** | negative | Neuropathy: Paraneoplastic etiology?  → No evidence for malignancy | Guillain Barré Syndrome,  DD: CIDP | TN |
| 17 | 61/M | 14 | **+** +MRI Spine | **-/-/-** | **-** | **-** | **+** | **+** | negative | Neuropathy: Paraneoplastic etiology?  → No evidence for malignancy | CIDP | TN |
| 18 | 51/F | 15 | **+** | **+/+/-** | **-** | **-** | **+** | **+** | negative | Suspected paraneoplastic etiology, malignancy?  → No evidence for malignancy | Facial palsy;  suspected inflammatory etiology | TN |
| 19 | 80/F | 9 | **+** | **+/+/+** | **-** | **+** | **+** | **-** | negative | Seizures, suspect thoracic lesion: malignancy?  → No evidence for malignancy | Inflammatory brain lesion  Paraneoplastic / metastatic etiology was excluded | TN |
| 20 | 35/F | 10 | **+*** *MRI Spine | **+/-/-** | **-** | **+** | **+** | **+** | negative | Myoclonus: malignancy?  → No evidence for malignancy | Symptomatic focal epilepsy | TN |
| 21 | 41/M | 45 | **-** | **+/+/+** | **-** | **-** | **+** | **+** | negative | Severe wasting: malignancy?  → Increased FDG uptake right apical lung | SAH, Critical illness PNP Neoplastic disease excluded Tuberculosis excluded | FP |
| 22 | 36/F | 18 | **+** | **+/+/+** | **+** | **+** | **+** | **-** | negative | Ischemic stroke, paraneoplastic coagulopathy? → suspected bronchioalveolar carcinoma in left lung | Ischemic stroke due to Paraneoplastic coagulopathy bronchoalveolar carcinoma | TP |

**Table B Patients with suspected large vessel vasculitis**

**Diagnostic tests before FDG-PET/CT, final diagnosi**s and correlation with FG-PET/CT:

| **Pat. No.** | **Age/ Sex** | **Days  on ICU** | **MRI Brain** | **CT: Brain/ Chest** | **CTA/MRA/ conventional Angiography** | **Vascular US:**  **TCD/Duplex** | **US:**  **TTE/TEE** | **Question addressed by FDG-PET/CT:**  **→Result** | **Confirmed Diagnosis** | **PET CT** |
| --- | --- | --- | --- | --- | --- | --- | --- | --- | --- | --- |
| 1 | 60/M | 47 | **+** | **+/+** | **-/+/-** | **-/+** | **+/+** | Large vessel vasculitis?  → No evidence for large vessel vasculitis | **Small vessel vasculitis** | TN |
| 2 | 60/F | 41 | **+** | **+/-** | **+/+/-** | **+/+** | **-/-** | Evidence for vasculitis activity under steroid therapy?  → No evidence for persisting vasculitis | Ischemic stroke left A. cerebri ant. and media **Primary CNS vasculitis** | TN |
| 3 | 53/M | 10 | **+** | **+/-** | **-/+/-** | **+/+** | **+/-** | Large vessel vasculitis?  → No evidence for large vessel vasculitis | Multiple ischemic strokes,  cerebral vasculitis | TN |
| 4 | 51/F | 19 | **+** | **+/-** | **+/-/-** | **+/+** | **+/-** | Evidence for vasculitis activity under immunosuppressive therapy?  → No evidence for persisting vasculitis | Seizures,  traumatic SAH,  inactive vasculitis | TN |
| 5 | 42/F | 15 | **+** | **+/-** | **-/+/+** | **+/+** | **-/+** | Evidence for vasculitis activity under steroid therapy?  → No evidence for persisting vasculitis | Takayasu arteritis,  Occlusion Truncus brachiocephalicus,  stroke right A. cerebri med. | TN |
| 6 | 63/F | 5 | **+** | **+/-** | **-/+/+** | **+/+** | **-/+** | Large vessel vasculitis?  → No evidence for large vessel vasculitis | Stroke right ACM,  **Suspected vasculitis** steroids initiated | TN |
| 7 | 38/F | 12 | **+** | **+/+** | **-/+/-** | **+/+** | **-/-** | Large vessel vasculitis?  → vasculitis of supraaortal branches | **Takayasu arteritis** | TP |
| 8 | 32/M | 11 | **+** | **+/-** | **-/+/-** | **+/+** | **+/-** | Large vessel vasculitis?  → No evidence for large vessel vasculitis | Multiple embolic strokes right ACM  Dissection ACI  Anti-Phospholipid AB syndrome | TN |
| 9 | 31/M | 11 | **+** | **+/-** | **-/+/+** | **+/+** | **+/-** | Large vessel vasculitis?  → No evidence for large vessel vasculitis | Multiple, embolic ischemic strokes right A. cerebri media  **Suspected vasculitis of middle sized vessels** | TN |
| 10 | 58/M | 8 | **+** | **+/-** | **+/+/-** | **+/+** | **+/-** | Large vessel vasculitis?  → No evidence for large vessel vasculitis | Ischemic strokes right A. cerebri media and anterior  Severe atherosclerosis | TN |
| 11 | 49/M | 14 | **+** | **+/-** | **-/+/-** | **+/+** | **-/+** | Large vessel vasculitis?  → Vasculitis of ascending aorta and Truncus brachiocephalicus | Multiple ischemic strokes right middle cerebral artery  Large vessel vasculitis  (**Takayasu Arteritis**) | TP |

**Table C Patients with suspected unknown infectious focus**

**Diagnostic tests before FDG-PET/CT, final diagnosis and correlation with FG-PET/CT:**

| **Pat. No.** | **Age/ Sex** | **Days on ICU** | **CSF** | **Cultures: R/B/U** | **MRI: Brain/Spine** | **CT scan:  Brain/ Chest/ Abdomen** | **US:**  **TTE/TEE/Abd** | **Question addressed by FDG-PET/CT:**  **→Result** | **Final diagnosis** | **Outcome** | **PET CT** |
| --- | --- | --- | --- | --- | --- | --- | --- | --- | --- | --- | --- |
| 1 | 88/M | 27 | **+** | **+/+/+** | **-/+** | **+/+/+** | **+/-/-** | Spondylodiscitis: further septic foci?  → Spondylodiscitis Th 10-12,  bilateral pneumonia (not demonstrated on ChX) | Spondylodiscitis,  bilateral pneumonia (MRSA) | died | TP |
| 2 | 69/M | 19 | **-** | **+/+/+** | **-/-** | **+/+/+** | **+/+/+** | Septic focus? Spondylodiscitis?  → Thrombophlebitis right forearm | Thrombophlebitis right forearm,  Heart insufficiency NYHA III | survived | TP |
| 3 | 62/M | 16 | **-** | **+/+/+** | **-/+** | **-/+/+** | **-/+/-** | Past spondylodiscitis, present pneumonia: further septic foci?  → bilateral pneumonia,  Exclusion of spondylodiscitis, no detection of further foci | Severe pneumonia Spondylodiscitis | survived | TN  (for further septic foci) |
| 4 | 80/M | 31 | **+** | **+/+/+** | **-/-** | **+/+/+** | **+/+/-** | Septic encephalopathy, septic focus?  → Endocarditis | Septic encephalopathy due to endocarditis | survived | TP |
| 5 | 57/M | 17 | **+** | **+/+/-** | **+/-** | **-/+/+** | **+/+/-** | Multiple cerebral abscesses due to Nocardiosis. Location of further abscesses?  → Detection of further absesses in the chest and abdomen | Nocardiosis | survived | TP |
| 6 | 72/M | 44 | **+** | **+/+/+** | **-/-** | **+/-/-** | **-/+/-** | Aseptic meningitis, inflammatory focus?  → Exclusion septic focus | Autoimmune disease | survived | TN |
